# Emerging Approaches in Pediatric Surgery: Current Evidence and Remaining Challenges

**DOI:** 10.3390/children13081023

**Published:** 2026-08-01

**Authors:** Donatella Di Fabrizio, Edoardo Bindi, Giovanni Cobellis

**Affiliations:** 1 Pediatric Surgery Unit, Salesi Children’s Hospital, 60123 Ancona, Italy; 2 Department of Specialized Clinical and Odontostomatological Sciences, University Politecnica of Marche, 60126 Ancona, Italy

## 1. Introduction

Pediatric surgery continues to incorporate minimally invasive techniques, image-guided procedures, artificial intelligence (AI), digital pathology, interventional radiology, and structured perioperative care. The assessment of these approaches requires more than technical feasibility. Their use in children must account for small anatomy, age-dependent physiology, rare and heterogeneous diseases, long life expectancy, and outcomes relevant to patients and families. The main question is whether a new approach improves diagnosis, operative decision-making, recovery, safety, or quality of life in a reproducible manner.

The 13 contributions included in the Special Issue “Advances in Pediatric Surgery: Innovative Approaches and Emerging Technologies” describe recent applications across several areas of pediatric surgery. They comprise original studies, reviews, a randomized clinical trial, and analyses of learning curves and high-risk conditions. The topics include fluorescence-guided surgery, vascular anomalies, AI-assisted imaging and pathology, minimally invasive and endovascular treatment, enhanced recovery, ambulatory surgery, and emergency multidisciplinary care [1,2,3,4,5,6,7,8,9,10,11,12,13]. The strength of the evidence varies across contributions, from preliminary case series to comparative studies. Common issues include patient selection, pediatric validation, standardized outcomes, and the integration of new methods into clinical workflows.

## 2. Image-Guided Surgery: Current Applications

The review by Iglesias et al. summarizes the reported uses of indocyanine green (ICG) across pediatric hepatobiliary, gastrointestinal, thoracic, lymphatic, urologic, gynecologic, plastic, otolaryngologic, ophthalmologic, neurosurgical, and oncologic procedures [2]. Route, dose, and timing determine which structures become visible and therefore require procedure-specific planning. Evidence remains uneven across indications, and fluorescence is still interpreted mainly as a qualitative signal. Further studies should standardize acquisition protocols and assess quantitative measures of perfusion, lymphatic flow, and tumor-to-background contrast.

Two studies examined ICG lymphography in children with lymphatic malformations. In a retrospective cohort of 83 children with macro- or mixed cystic lymphatic malformations, Han et al. reported an association between intracystic hemorrhage and a more favorable therapeutic outcome [1]. Intraoperative ICG lymphography showed an absence of afferent lymphatic vessels in hemorrhagic lesions, which the authors proposed as a possible explanation. A randomized clinical trial from the same group compared ICG lymphography-guided partial resection plus penetration sclerotherapy with conventional partial resection plus sclerotherapy in 39 children with microcystic lymphatic malformations [11]. The cure rate was higher in the guided group, 65.0% versus 26.3%, with no significant differences in operative time or complications. The modest sample size and limited follow-up support cautious interpretation and further validation.

Taken together, these studies suggest that intraoperative imaging is most relevant when linked to a defined action, such as determining the extent of resection, identifying residual areas for treatment, or assessing perfusion. Future trials should prespecify how fluorescence findings influence surgical decisions and should evaluate recurrence, functional outcomes, repeated procedures, and measurement consistency across cameras, doses, institutions, and patient sizes.

## 3. Management of Vascular Anomalies

Guida et al. reported an initial prospective experience with bleomycin electrosclerotherapy for peripheral low-flow venous and lymphatic malformations [3]. The series included 12 children. Reductions in lesion dimensions were observed, and no relevant post-procedural complications were reported. The technique combines a sclerosing agent with electroporation to increase local cellular uptake, with the aim of improving local treatment while limiting systemic exposure.

This study provides preliminary feasibility and safety data but does not establish comparative effectiveness or long-term durability. Larger studies should apply standardized classification, imaging-based volumetry, symptom scores, cosmetic and functional assessment, patient-reported outcomes, and longer surveillance. Collaborative registries would improve comparisons among sclerotherapy, electrosclerotherapy, systemic targeted therapy, surgery, and combined approaches while accounting for lesion type, anatomical extent, and previous treatment.

## 4. Artificial Intelligence and Digital Diagnostics

AI was examined in two different clinical settings. Simmler et al. compared automated and manual radiographic measurements after subtalar arthroereisis for symptomatic flexible flatfoot [6]. Agreement was satisfactory for several dorsoplantar angles, but systematic differences emerged for selected lateral-view parameters, and the software did not analyze a substantial proportion of radiographs. Image acquisition quality did not consistently prevent the system from generating measurements. These results indicate that automated outputs still require technical and clinical review.

Di Fabrizio et al. evaluated multimodal large language models applied to ex vivo fluorescence confocal microscopy in 20 pediatric surgical cases [8]. Both systems identified all 13 neoplastic cases, while errors occurred mainly in non-neoplastic lesions that resembled tumors. Adding immunohistochemical information improved performance in the paired subgroup of five cases and resolved false-negative classifications. Given the small and heterogeneous cohort, the findings represent proof-of-concept evidence. At present, the appropriate role of these systems is decision support within a pathologist-led workflow.

Further pediatric surgical AI studies should prioritize external and prospective validation across institutions, devices, acquisition protocols, age groups, and disease prevalence. Evaluation should report failed analyses, calibration, class-specific errors, uncertainty, interobserver comparison, and the clinical consequences of incorrect outputs. Studies should also assess effects on decisions, diagnostic time, workflow, and patient outcomes. Data governance, privacy, model updating, and human oversight require explicit definition. Because pediatric datasets are often small, multicenter collaboration and transparent reporting are particularly important.

## 5. Less Invasive Care and Perioperative Pathways

Behrmann et al. assessed an enhanced recovery after surgery protocol in children undergoing thoracoscopic lung resection [9]. In a retrospective comparison of 10 ERAS patients and 10 historical controls, protocol use was associated with shorter hospital stay and chest-tube duration, lower opioid exposure and costs, and fewer major complications. These findings support the further evaluation of standardized anesthesia, analgesia, tube management, feeding, and discharge pathways. The small cohort and retrospective design do not permit firm conclusions regarding safety or effect size.

Delgado–Miguel et al. examined outpatient surgery for adolescent gynecomastia without closed-suction drains [7]. All 21 patients were discharged on the day of surgery, and two mild seromas resolved without intervention. The series indicates feasibility in selected patients, but the absence of a comparison group limits conclusions about the specific effect of omitting drainage. Comparative studies should include satisfaction, pain, body image, return to daily activities, and other patient-reported outcomes in addition to postoperative complications.

Training and proficiency are another aspect of implementation. Yang analyzed 469 laparoscopic intracorporeal sac transections with purse-string repair for pediatric inguinal hernia using cumulative sum analysis [12]. Operative time improved after approximately 233 cases, while recurrence remained low during both phases. The study distinguishes operative efficiency from safety, although the threshold identified in a single-surgeon series should not be assumed to apply to all surgeons or training settings. Multi-surgeon studies should examine risk-adjusted outcomes and whether simulation, video review, coaching, or structured assessment shortens the learning process.

These contributions indicate that evaluation of less invasive care should extend beyond the procedure itself. Relevant outcomes include pain, opioid exposure, recovery time, unplanned contact, readmission, cost, family burden, and long-term function. Protocol adherence and reasons for deviation should also be reported to distinguish the effects of an intervention from those of its implementation.

## 6. Endovascular Approaches in Selected High-Risk Settings

Losin et al. described endovascular embolization in six children with pulmonary sequestration and contraindications to surgery, including neonates with major cardiopulmonary comorbidity [13]. Technical success was achieved in all patients, no major procedural complications occurred, and follow-up showed regression of the sequestered tissue, hemodynamic improvement, and no subsequent resection. The series supports embolization as an option in selected high-risk patients, but its size and non-comparative design do not establish equivalence to surgery or support routine use. Parallel cohorts and prospective registries are needed to define indications, device selection, surveillance, reintervention, and long-term outcomes in growing children.

Di Mitri et al. addressed aorto-esophageal fistula after foreign-body ingestion [10]. Their report of covered-stent placement in a hemodynamically unstable child was accompanied by a review of 36 published pediatric cases. Button batteries accounted for 67% of cases, and overall mortality was 43% [10]. The case illustrates endovascular control as a possible emergency or bridging option, while the available evidence remains limited to case reports and small series. International registries with agreed data elements are needed to clarify management. Prevention, recognition of sentinel bleeding, computed tomography angiography when vascular injury is suspected, and coordinated multidisciplinary care remain central.

## 7. Clinical Decision-Making with Limited Evidence

Pogorelić et al. reviewed salivary biomarkers for pediatric acute appendicitis [4]. Only three biomarkers had been studied in fewer than 300 children. Salivary C-reactive protein showed favorable diagnostic estimates in the available studies, while leucine-rich alpha-2-glycoprotein 1 and irisin had limitations as standalone tests. Before clinical use, salivary biomarkers require multicenter validation, standardized sampling, age-specific reference values, and prospective assessment within existing clinical and ultrasound pathways. Multimarker point-of-care panels warrant evaluation, including their effect on imaging use, diagnostic delay, and negative appendectomy.

Xu et al. evaluated decompressive craniectomy for severe pediatric traumatic brain injury using a matched retrospective cohort [5]. Surgery was not associated with a significant reduction in in-hospital mortality or resource use. Neurological status was better at discharge, while the difference at three months was not statistically significant. Residual confounding remains possible despite matching, and the results do not establish a causal benefit. More consistent evidence will require standardized indications, longer neurodevelopmental follow-up, harmonized outcome definitions, and prospective multicenter data collection.

## 8. Conclusions

This Special Issue presents a varied set of recent developments in pediatric surgery, including fluorescence-guided procedures, AI-assisted diagnostics, endovascular treatment, enhanced recovery, and ambulatory care. The contributions provide useful clinical data and identify areas for further study, but many findings arise from small, single-center, retrospective, or non-comparative cohorts. They should therefore be viewed as preliminary or context-specific where appropriate. Future research should focus on multicenter validation, standardized outcomes, longer follow-up, and the direct assessment of whether a new method improves clinical decisions or patient outcomes. Pediatric surgeons, engineers, radiologists, pathologists, anesthesiologists, intensivists, nurses, and families all have relevant roles in the design and evaluation of these approaches. Careful collaboration should help distinguish methods suited for broader clinical adoption from those that still require further assessment.
